# Chagas disease: host responses, parasite evasion and vaccine advances

**DOI:** 10.1093/trstmh/traf109

**Published:** 2025-10-06

**Authors:** Héctor Serrano-Coll

**Affiliations:** Tropical medicine, Instituto Colombiano de Medicina Tropical-Universidad CES, Cra. 43A #52 Sur - 99, Medellín 055450, Colombia

**Keywords:** access to vaccine, immune evasion, immune response, *Trypanosoma cruzi*, vaccine development

## Abstract

Chagas disease is a parasitic infection caused by *Trypanosoma cruzi*. Although the host immune response has been extensively studied, important knowledge gaps remain, particularly regarding the complex and multifaceted immune evasion mechanisms employed by the parasite, both at the innate and adaptive levels. In recent years, multiple promising vaccine candidates have been developed, but further evaluation is needed across the different phases of clinical trials. Therefore, the aim of this review is to examine the host immune response to *T. cruzi*, the parasite’s immune evasion strategies and recent advances in vaccine development aimed at controlling infection.

## Introduction

**Figure 1.**
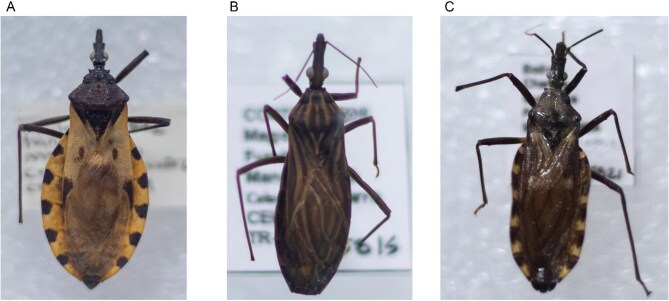
Triatomine bugs. (A) Adult *Triatoma dimidiata*, exhibiting fully developed wings. Specimen code: CEICMT-5842; collected in Colombia, Huila Department, Tarquí Municipality. (B) Nymphal stage of *Rhodnius prolixus*, lacking wing development. Specimen code: CEICMT-5815; collected in Colombia, Magdalena Department, Fundación Municipality. (C) Adult *Triatoma infestans*, with well-developed and visible wings. Specimen code: CEICMT-5821; collected in Bolivia, Potosí Department. All specimens are part of the entomological collection of the Instituto Colombiano de Medicina Tropical (ICMT).

Chagas disease, or American trypanosomiasis, is a neglected infectious disease caused by *Trypanosoma cruzi*.^1^ It is primarily transmitted to humans and animals by hematophagous triatomine insects of the Reduviidae family, such as *Triatoma infestans, Rhodnius prolixus* and *Triatoma dimidiata*.^2^ Figure 1 shows triatomine bugs. These insect vectors play a central role in disease transmission, which occurs when *T. cruzi* is excreted in the insect’s feces during or after feeding and enters the host through broken skin or mucosal surfaces such as the conjunctiva.^3^ In addition to vector-borne transmission, other important infection routes include ingestion of contaminated food or beverages (oral transmission),^4^ vertical (congenital) transmission and transmission via blood transfusion or organ transplantation.^5,6^ Although less commonly recognized, *T. cruzi* can also be transmitted via occupational exposures, such as laboratory accidents involving infected material.^7^

Following infection, Chagas disease typically progresses through two clinical phases: an acute phase (often asymptomatic or mild) and a chronic phase. While most chronically infected individuals remain asymptomatic for life (the indeterminate form), a significant subset eventually develops life-threatening cardiac or gastrointestinal complications.^8,9^

Epidemiologically, Chagas disease remains endemic in Latin America, where approximately 70 million people are at risk of infection.^10^ An estimated 6–7 million individuals are currently infected with *T. cruzi*,^11^ with the highest prevalence reported in Bolivia, followed by Argentina, Brazil and Mexico.^12^ However, patterns of human migration and the effects of climate change have contributed to the emergence of Chagas disease as a global public health concern.^13,14^

Despite decades of research, gaps in knowledge persist, particularly in the understanding of host–parasite immune interactions, which are critical for designing effective interventions. As no licensed vaccine currently exists, and current treatments are limited in efficacy and tolerability, advancing our understanding of the immune response to *T. cruzi* and the parasite’s immune evasion strategies is essential. In this context, this review explores the innate and adaptive immune responses to *T. cruzi*, highlights the mechanisms the parasite uses to evade host defenses and discusses recent progress in the development of vaccines for Chagas disease.

## Life cycle of *Trypanosoma cruzi*

When a triatomine (vector) feeds on the blood of an infected host, it ingests trypomastigotes present in the bloodstream.^15^ These parasites travel to the vector’s stomach, where they transform into spheromastigotes. Then, in the midgut of the insect, they develop into epimastigotes, which actively multiply by binary fission.^16^ Subsequently, the epimastigotes migrate to the hindgut and the rectal ampulla of the triatomine, where they differentiate into metacyclic trypomastigotes, which are the infective forms for mammals.^16^

When the infected vector bites a new host (human or animal), it defecates near the bite site.^17^ The feces contain metacyclic trypomastigotes, which can enter the host through eroded or broken skin, mucous membranes such as the conjunctiva of the eye, or via the oral route by consuming contaminated food (such as fruits, vegetables or other items) containing vector feces.^11,17,18^ Once inside the host, the trypomastigotes invade resident macrophages or other nucleated cells at the site of inoculation.^19^ Inside the cell, they transform into amastigotes, which actively multiply by binary fission.^19^

Later, the amastigotes transform again into trypomastigotes, which lyse the host cells and disseminate through the blood and lymphatic system, infecting new tissues and repeating the cycle.^19^

## Innate and adaptive immune response against *Trypanosoma cruzi*

The immune response to *T. cruzi* involves a coordinated interplay between innate and adaptive mechanisms that contribute to parasite control, although complete sterile immunity is rarely achieved.

## Innate immune response

**Figure 2.**
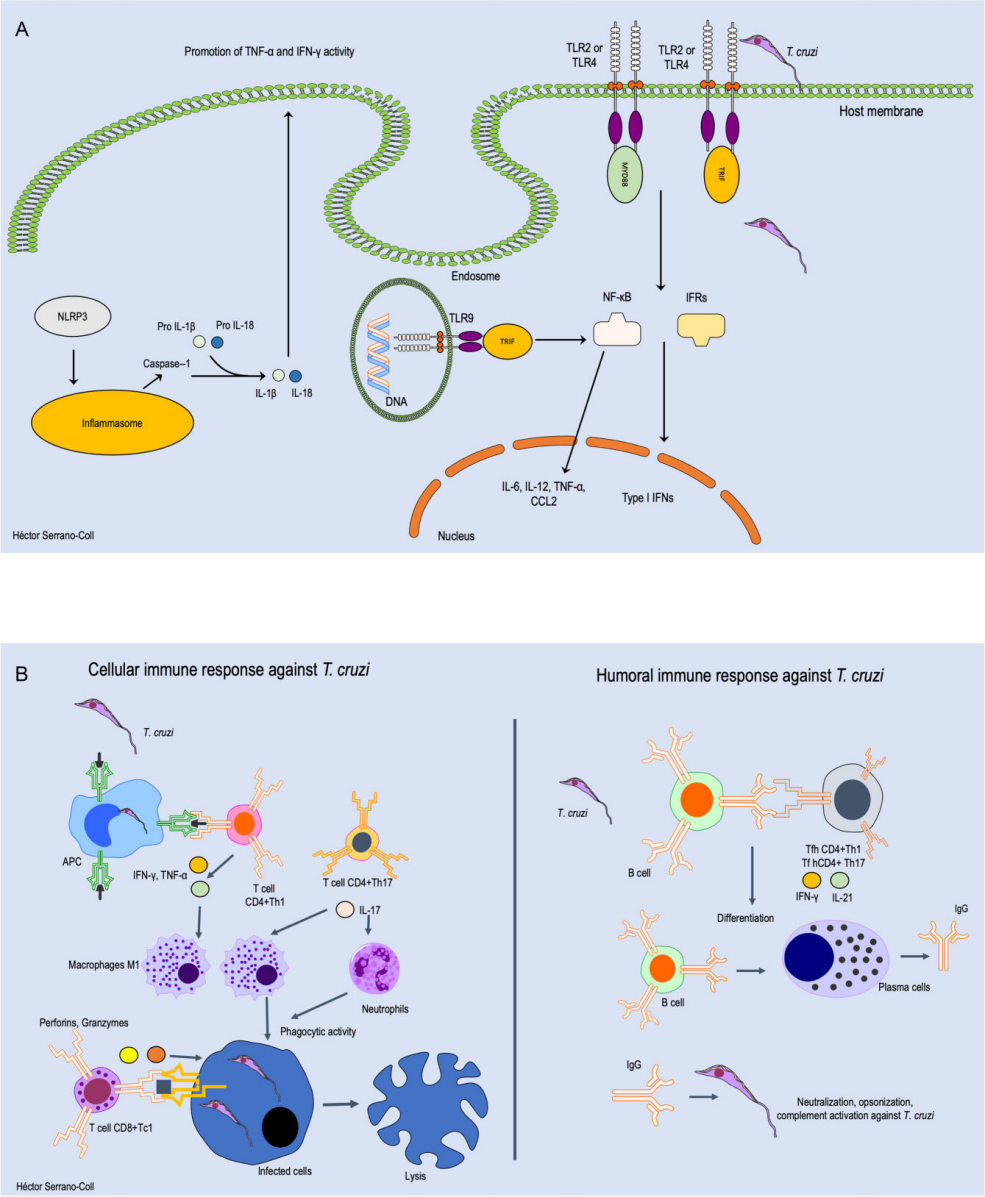
Innate and adaptive host mechanisms against *T. cruzi*. (A) This figure shows innate mechanisms such as TLRs 2, 4 and 9 recognizing *T. cruzi* PAMPs or DNA, which can activate both MyD88-dependent and -independent pathways against this parasite, as well as the involvement of inflammasome activation leading to the release of IL-1β and IL-18. (B) The adaptive immune response driven by a Th1 and Th17 effector pattern, which induces a cellular immune response against *T. cruzi* and promotes the differentiation of B lymphocytes into IgG-producing plasma cells. APC: antigen-presenting cell; PAMPs: pathogen-associated molecular patterns; TLRs: Toll-like receptors. Parts of images from Motifolio drawing toolkits (Motifolio, Inc.) were utilized in the figure preparation.

Innate immunity plays a central role during the acute phase of *T. cruzi* infection, acting as the first line of defense. This response relies on the recognition of pathogen-associated molecular patterns by pattern recognition receptors (PRRs), triggering downstream signaling pathways that initiate inflammation and parasite control.^20^ Among these, Toll-like receptors (TLRs) are key players. Specifically, TLR2, TLR4 and TLR9 recognize parasite components such as glycosylphosphatidylinositol (GPI) anchors, surface lipoproteins and unmethylated CpG-rich DNA, respectively.^21,22^ Upon ligand binding, these receptors recruit the adaptor protein MyD88, leading to NF-κB activation and the induction of pro-inflammatory cytokines like IL-6, IL-12 and TNF-α, as well as chemokines such as CCL2 and adhesion molecules that support immune cell recruitment.^23^ Additionally, TLRs can activate a MyD88-independent signaling pathway when TLR3 and TLR4 recruit the adaptor protein TRIF, leading to the induction of type-I interferons, primarily IFN-β.^24^ These cytokines, in turn, activate natural killer (NK) cells,^25^ which identify infected cells with downregulated MHC class-I expression and eliminate them through perforin and granzyme-mediated cytotoxicity.^26^ Figure 2A describes the innate immune response against *T. cruzi*.

NOD1, a cytosolic PRR, also contributes to innate detection of *T. cruzi*.^27^ Its activation recruits RIP2 kinase, triggering NF-κB and promoting the expression of pro-IL-1β and pro-IL-18.^28^ These cytokines are processed by caspase-1 via the NLRP3 inflammasome, yielding active IL-1β and IL-18,^29^ which enhance the production of IFN-γ and TNF-α, key mediators in parasite control.

The complement system also contributes to innate defense against *T. Cruzi* and can be activated through the lectin and alternative pathways, independently of antibodies.^30^ Parasite surface sugars can be recognized by mannose-binding lectin (MBL), or spontaneous C3b deposition can occur.^30^ Additionally, antibodies bound to the parasite surface can activate the classical pathway.^30^ These mechanisms result in the opsonization of the parasite by C3b, the recruitment of macrophages and neutrophils through anaphylatoxins such as C3a and C5a, leading to parasite clearance either by phagocytosis or through the release of neutrophil extracellular traps, as well as the formation of the membrane attack complex (MAC), ultimately causing direct lysis of the parasite.^23,31^

## Adaptive immune response

The adaptive immune response is initiated during the acute phase of *T. cruzi* infection, when antigen-presenting cells (APCs) process parasitic antigens and present them to naïve CD4⁺ T cells via major histocompatibility complex class II (MHC-II) molecules. This interaction drives the differentiation of CD4+ T cells into two main effector subsets: Th1 and Th17 cells.^32,33^ Th1 cells primarily produce IL-2, TNF-α and IFN-γ,^34^ with IL-2 being critical for the clonal expansion of both CD4+ and CD8+ T lymphocytes.^35,36^ In addition, IFN-γ enhances macrophage polarization toward a pro-inflammatory M1 phenotype, characterized by increased microbicidal and phagocytic activity.^34^ Moreover, Th17 cells also contribute significantly to parasite control by secreting IL-17, IL-21 and IL-23, which sustain inflammatory responses and promote the recruitment of neutrophils to sites of infection.^34^ TNF-α and IL-17, in particular, play key roles in the mobilization of phagocytic cells, aiding in the containment and elimination of the parasite. In parallel, CD8+ T cells recognize *T. cruzi*-derived peptides presented on MHC-I molecules and differentiate into cytotoxic Tc1 cells, which are capable of directly lysing infected host cells.^23,37^ Together, these coordinated cellular responses form the backbone of the adaptive immune defense against *T. cruzi*. Figure 2B describes the adaptative immune response against *T. cruzi*.

Concurrently, B lymphocytes recognize *T. cruzi* antigens via their B cell receptors, internalize them and present processed peptides via MHC II to helper T cells.^23,38^ Engagement of costimulatory molecules such as CD40-CD40L and ICOS-ICOSL fosters the differentiation of CD4+ T cells into follicular helper T cells (Tfh).^23^ Initial T-B interactions lead to the generation of short-lived plasma cells secreting IgM.^39^ However, a subset of B cells migrates to germinal centers within secondary lymphoid organs, where, under the influence of IL-21 secreted by Tfh cells, they undergo clonal expansion, somatic hypermutation and class-switch recombination, ultimately differentiating into plasma cells that produce high-affinity IgG antibodies against *T. cruzi*.^38^ These antibodies mediate classical complement activation and contribute to parasite destruction; however, the most important complement pathways involved in the immune response against *T. cruzi* are the lectin and alternative pathways.^31^

Furthermore, CD8+ Tc1 cells recognize *T. cruzi*-infected host cells via antigen presentation on MHC-I molecules.^40^ Upon activation, they release perforin and granzymes, inducing apoptosis of the infected cells and limiting intracellular replication of the parasite.^41^

## Immune evasion by *T. cruzi* in the host

It is essential to understand why, despite the activation of both innate and adaptive immune responses described in the previous section, these mechanisms often fail to prevent the establishment and progression of *T. cruzi* infection. An overview is provided of the major immune evasion strategies employed by *T. cruzi* to circumvent host defenses and promote its survival and long-term persistence.

## Innate immune evasion

**Figure 3.**
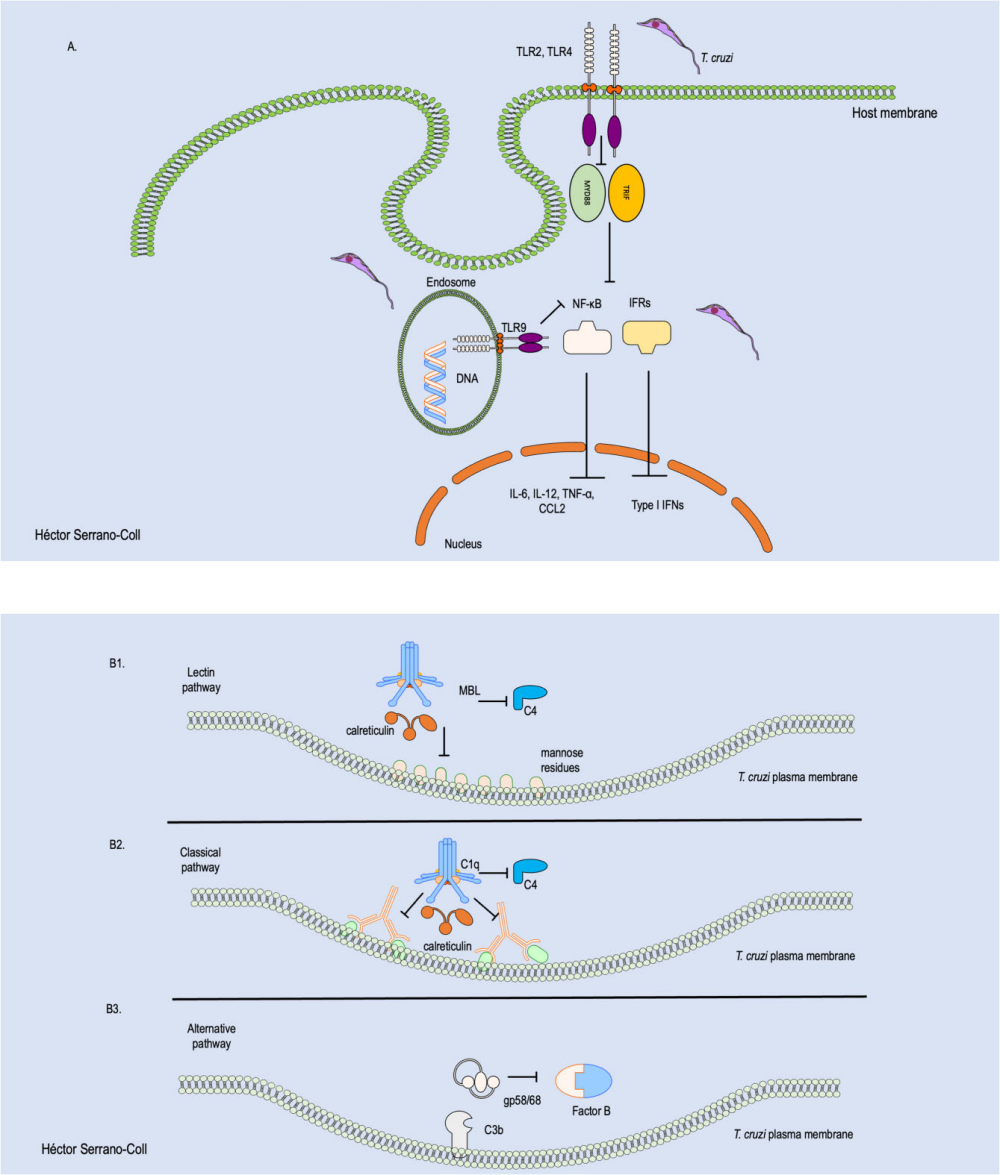
Mechanisms of innate immune evasion by *T. cruzi*. (A) How *T. cruzi* induces the inhibition of adaptor protein recruitment TRIF and MyD88 at TLRs 2, 4, and 9, thereby preventing the release of type I IFNs and pro-inflammatory cytokines. (B) How proteins such as calreticulin block the classical and lectin pathways, preventing the formation of the C3 convertase (B1, B2). In the alternative pathway, this inhibition of C3 convertase formation is mediated by the GP58/68 protein (B3). TLRs: Toll-like receptors. Parts of images from Motifolio drawing toolkits (Motifolio, Inc.) were utilized in the figure preparation.

*Trypanosoma cruzi* has developed multiple sophisticated strategies to evade the host’s innate immune response, particularly TLRs.^20^ One of the main tactics involves negative modulation of the interaction between the TIR domains of TLRs and their intracellular adaptor proteins, such as MyD88 and TRIF, thereby impairing both the MyD88-dependent and -independent signaling pathways.^42^ This blockade partially inhibits the activation of key transcription factors such as NF-κB and interferon regulatory factors (IRFs), which are crucial for the production of pro-inflammatory cytokines required to initiate an effective cellular immune response.^23^ Furthermore, inhibition of IRFs via the MyD88-independent pathway results in reduced expression of type I interferons, which are critical for the activation of NK cells,^43^ thus favoring parasite survival within the host. Figure 3A illustrates the mechanisms employed by *T. cruzi* to evade recognition by TLRs.

Further enhancing its evasion, *T. cruzi* promotes the release of extracellular vesicles (EVs) from infected macrophages. These EVs interact with TLR2 and trigger partial signaling that induces the production of cytokines such as TNF-α, IL-6 and IL-1β, sustaining a low-grade inflammatory response that is insufficient to eliminate the parasite.^44^ The parasite also exploits TLR2 to facilitate its entry into macrophages by activating Rab5, thereby avoiding immediate immune detection.^45^ Moreover, activation of TLR4 and TLR9 can be attenuated by structural modifications in parasitic ligands, such as GPI anchors and unmethylated CpG motifs in DNA,^45,46^ respectively, or by host genetic polymorphisms, such as the TLR4 299Gly/399Ile haplotypes, which reduce receptor sensitivity.^47^ Altogether, these strategies allow *T. cruzi* to precisely manipulate TLR signaling, limiting effective immune activation while avoiding immune-mediated clearance.

Another mechanism employed by *T. cruzi* to evade the immune response is the inhibition of the complement system through its three pathways: classical, alternative and lectin. This inhibition is primarily achieved by blocking the formation of the C3 convertase, a key step in the activation of this proteolytic cascade.^30^ When the C3 convertase is not formed, there is no release of anaphylatoxins, no opsonization by C3b and no formation of the MAC, all of which contribute to the parasite’s survival.^48^ Figure 3B illustrates the mechanisms employed by *T. cruzi* to evade the activity of the complement system.

The blockage of C3 convertase in the classical and lectin pathways is mediated by a multifunctional 45 kDa protein located mainly on the surface of *T. cruzi*, called calreticulin (TcCRT). In the lectin pathway, TcCRT binds to the MBL and L-ficolin, preventing its interaction with sugars on the parasite surface.^49^ As a result, the recruitment and cleavage of C4 are blocked, which inhibits the initiation of C3 convertase formation.^49^ In the classical pathway, the same protein binds to C1q, preventing it from recognizing at least two Fc regions of antibodies. Consequently, this pathway is not activated, C4 is neither recruited nor cleaved and the C3 convertase is not formed.^49^

In the alternative pathway, another surface protein from *T. cruzi* known as gp58/68 plays a role in immune evasion.^50^ This glycoprotein interferes with the binding of factor B to C3b, effectively preventing the formation of the C3 convertase (C3bBb) specific to this pathway.^50,51^

## Adaptative immune evasion

**Figure 4.**
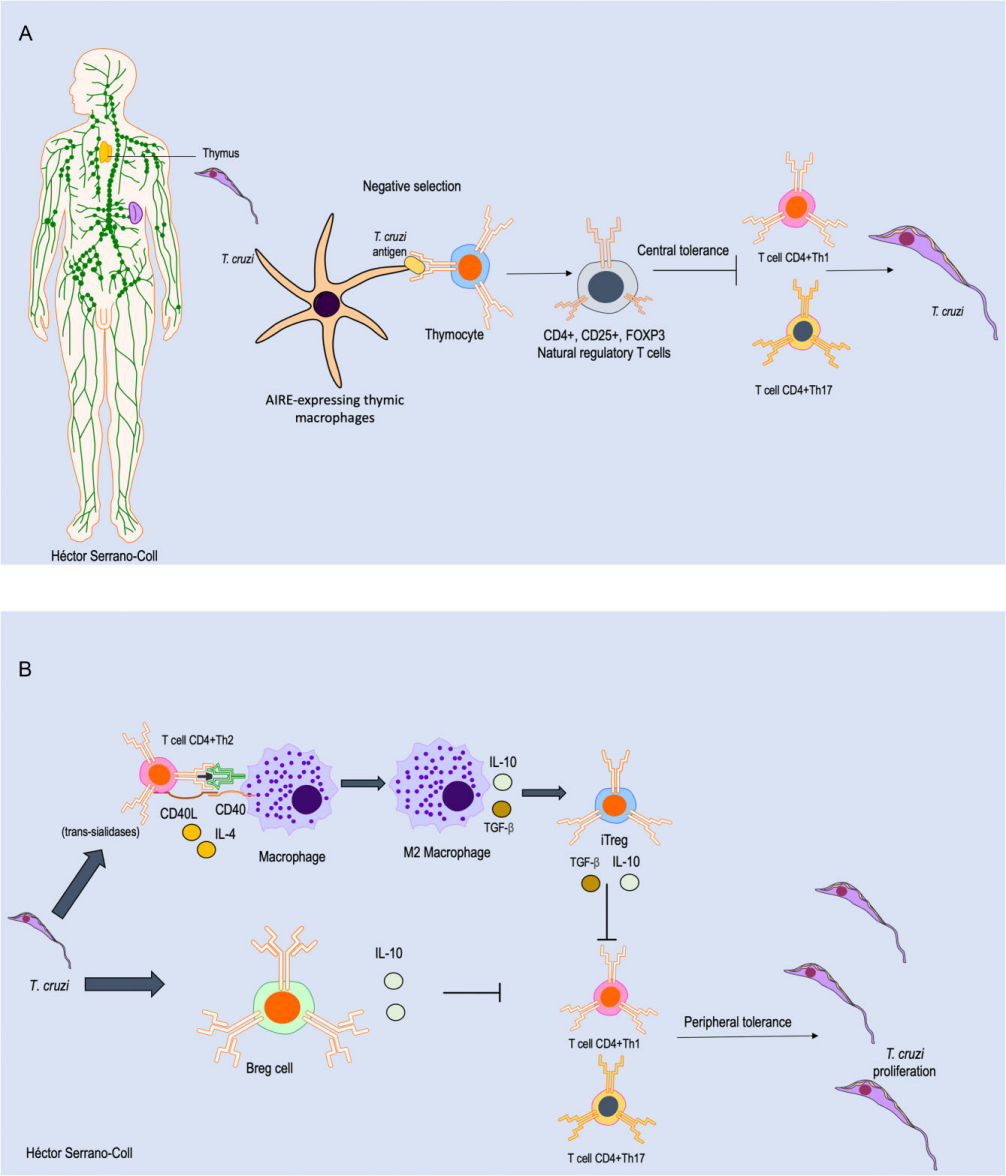
Mechanisms of adaptive immune evasion by *T. cruzi*. (A) Central tolerance: *T. cruzi* colonizes the thymus, promoting the development of natural regulatory T cells, which suppress CD4⁺ T cell-mediated Th1 and Th17 responses. (B) Peripheral tolerance: a Th2-skewed immune response leads to the generation of induced regulatory T cells (iTreg) and B regulatory B cells (Breg), which further inhibit Th1 and Th17 responses, aiding in immune evasion by *T. cruzi*. AIRE: autoimmune regulator. Parts of images from Motifolio drawing toolkits (Motifolio, Inc.) were utilized in the figure preparation.

From an adaptive perspective, *T. cruzi* can alter the neuroendocrine-immune axis by stimulating glucocorticoid production, which induces thymic atrophy primarily through apoptosis of CD4⁺CD8⁺ thymocytes, mediated by caspases 8 and 9.^52^ In parallel, *T. cruzi* may trigger the intrathymic expression of its own antigens, leading thymocytes present in both children and adults to recognize these antigens as self during negative selection in the thymic medulla.^53^ This process promotes central tolerance to the parasite by generating CD4⁺ and CD8⁺ T cells that are tolerant to *T. cruzi* antigens. Furthermore, it contributes to the development of natural regulatory T cells (CD4⁺, CD25⁺, FOXP3⁺), which suppress Th1 and Th17 responses by inhibiting the differentiation of naïve T cells into these effector phenotypes.^52,53^ In addition, cruzipain promotes a Th2-skewed immune response, characterized by elevated levels of IL-4, IL-5 and IL-10, which further impairs effective parasite clearance. It also induces alternative macrophage activation through the arginase pathway, reducing nitric oxide production, which is a critical mechanism for parasite killing.^54^ Together, these strategies create a permissive immunological environment that facilitates *T. cruzi* survival, dissemination and long-term persistence within the host.^48^ Figure 4A illustrates the mechanisms employed by *T. cruzi* to promote central tolerance.

Beyond central tolerance, *T. cruzi* can also induce peripheral immune tolerance, modulating the immune environment to avoid elimination and promote its long-term persistence within the host. Somoza et al.^38^ demonstrated that the parasite induces differentiation of B cells into a regulatory phenotype (Bregs) through a combination of innate signals (TLRs), costimulatory signals (CD40L) and autocrine factors (BAFF/APRIL). These Bregs are characterized primarily by IL-10 production, a cytokine that suppresses Th1 and Th17 responses by inhibiting APC activation and reducing pro-inflammatory cytokine production.^55,56^ This creates a tolerant immune environment that limits protective responses, allowing parasite replication, persistence and chronic disease development. Figure 4B illustrates the mechanisms employed by *T. cruzi* to promote peripheral tolerance.

Additionally, certain parasite virulence factors, such as trans-sialidases (TSs), promote the differentiation of CD4+ T cells toward a Th2 phenotype.^57^ This phenotype, characterized by the release of IL-4, induces a humoral response dominated by IgE, which has limited capacity for neutralization and opsonization key processes in the destruction of intracellular pathogens.^23^ Furthermore, it induces macrophage differentiation into the M2 or alternative phenotype, which promotes an anti-inflammatory environment with the release of IL-10 and predominantly TGF-β, both playing an important role in the generation of induced regulatory T cells (iTregs).^58^ This expansion of iTregs is also promoted by a major surface glycoprotein SSP4 of *T. cruzi.*^58^ These cells play a crucial role in reducing IL-2, which is essential for the clonal expansion of CD4+ and CD8+ T lymphocytes,^59^ and they inhibit the differentiation of CD4+ T cells into Th1 and Th17 phenotypes, which are key in infection control.

## Vaccines against *Trypanosoma cruzi*

Vaccination against *T. cruzi* is essential for controlling Chagas disease in the Americas and will be key to reducing both the incidence and the morbidity and mortality associated with this infection. In this context, the use of diverse technological approaches and vaccine platforms is critical to achieving effective control. The following section outlines some of the most promising vaccine strategies currently in the preclinical phase, which have shown encouraging results against *T. cruzi*.

## Attenuated vaccines using CRISPR-Cas9

CRISPR/Cas9 technology enables the precise deletion of key virulence genes in *T. cruzi*, allowing the development of safer and more stable live attenuated vaccines.^60,61^ These genetically modified strains retain their immunogenicity and can induce long-lasting protective immune responses against Chagas disease.^60^ The following section describes some vaccine candidates developed using this technology.

In a murine model using CRISPR-Cas9, Burle-Caldas et al.^61^ studied the role of Active Trans-Sialidase (aTS) of *T. cruzi*, demonstrating that this enzyme plays a critical role in the egress of the parasite from infected host cells. They observed that splenocytes from BALB/c mice immunized with aTS knockout (KO) parasites produced strong immune responses, suggesting that these immune cells may help prevent the propagation of infection and contribute to the development of effective vaccines. Notably, mice inoculated with the TS KO7 mutant showed limited infection, which was associated with increased IFN-γ production. This cytokine may promote macrophage polarization toward an M1 phenotype, enhancing their ability to phagocytose and eliminate parasites, thereby preventing the spread of infection. Therefore, TS KO mutants act as live attenuated vaccines capable of inducing protective immunity without causing disease. Disruption of aTS thus represents a promising strategy for the development of prophylactic vaccines against Chagas disease by generating a strong and long-lasting immune response without the risk of systemic infection. Table 1 describes vaccine candidates against *T. cruzi*.

**Table 1.** Vaccine candidates against *Trypanosoma cruzi*

	Vaccines against *Trypanosoma cruzi*						
Platform	Vaccine candidate	Antigen function	Immunogenicity	Experimental model	Advantages	Disadvantages	References
Attenuated vaccines using CRISPR-Cas9	aTS KO	aTS is critical for parasite egress from infected host cells	Limits infection spread via IFN-γ-driven M1 macrophage activation	Murine model	Precise deletion of virulence genes for safetyInduces strong, long-lasting immunity	Regulatory and safety concernsComplex production	^61^
	Cyp19 double-knockout *T. cruzi* strain	Cyp19 is essential for parasite replication, differentiation and dissemination within host cells	Th1-biased immunity (↑IFN-γ, ↓IL-4/IL-10), enhances IgG response	Murine model	Mimics natural infection closely		
DNA vaccines	Chagasin DNA vaccine delivered via *Salmonella enterica*	Cruzipain is essential for invasion, immune evasion and immunogenicity	↑IFN-γ, robust immune response, ↓parasite burden, ↓tissue damage	Murine model	Induces both humoral and cellular immunityStable and easy to produce	Requires multiple doses	^65^
Recombinant protein vaccines	TSA-1-C4* and Tc24-C4**	TSA-1 and Tc24 play roles in host cell invasion and immune evasion	Induces Th17/Tc1 responses, promotes IgG1–IgG3 switching and activates macrophages (↑NO, H₂O₂, IL-6, TNF-α)	*Murine model ** phase I clinical trials in humans	Targeted immune responses with specific antigensGood safety profileCan induce balanced Th1/Th2 responses	Production can be costly	^67–69^
	Multiepitope recombinant protein targeting HLA-A02:01-restricted *T. cruzi* epitopes	HLA-A02:01 presents conserved epitopes that promote CD8⁺ T cell-mediated cytotoxicity	Induces strong CD8⁺ Tc1 responses (↑IFN-γ), activates cytotoxic pathways (perforins, granzymes) and promotes Th1-driven IgG antibody production	Ex vivo PBMCs isolated from patients with Chagas disease			
	TcTASV recombinant + baculovirus formulation	TcTASV antigens mediate early host–parasite interaction and immune evasion; stimulate protective cellular and humoral immunity	Induces Th1 response (↑IFN-γ, IgG2a), activates CD8⁺ and CD4⁺ T cells, and produces lytic antibodies	Murine model			
RNA vaccines	mRNA-Tc24/ASP-2	Tc24: flagellar calcium-binding protein; promotes host cell invasion and strong CD4⁺/CD8⁺ T cell responsesASP-2: surface protein expressed in the amastigote stage; essential for intracellular survival and elicits strong CTL responses	Balanced Th1/Th2 cytokine response; promotes parasite control and durable memory	Murine model	Rapid design and production, strong and durable immune responses	Stability and storage challenges; higher production costs; limited data in parasitic infections	^71^

ASP-2: amastigote surface protein-2; aTS: active Trans-Sialidase; CTL: cytotoxic T lymphocyte; Cyp19: cyclophilin-19; KO: knockout; PBMCs: peripheral blood mononuclear cells; Tc24: calcium-binding protein 24; TcTASV: *T. cruzi* trypomastigote alanine, serine and valine-rich protein; TSA-1: trypomastigote surface antigen-1. *indicates the vaccine candidate TSA-1-C4, **correspond to Tc24-C4.

Another promising candidate using this technology is the double-knockout (DKO) strain of *T. cruzi*, in which two genes encoding cyclophilin 19 (Cyp19) have been deleted. Cyp19 is a key protein involved in parasite replication within host cells and in its ability to differentiate and disseminate.^62^ In murine models, inoculation with the DKO strain induces robust immune responses characterized by T cell activation and strong IFN-γ production, skewing the immune response toward a Th1 profile rather than Th2, as evidenced by low levels of IL-4 and IL-10, and this Th1 bias plays an important role in the generation of IgG antibodies against the parasite.^62^ Our hypothesis regarding this candidate is that, although the antibodies generated may not be sufficiently neutralizing due to the attenuated nature of the whole-parasite immunization, the induction of a strong Th1 and potentially Th17 immune response could be critical for reducing parasitic load and preventing progression to the chronic phase of Chagas disease. Therefore, this approach holds promise as a prophylactic strategy to control infection and mitigate long-term tissue damage associated with chronic Chagas disease.

## DNA vaccines

DNA vaccines introduce a small segment of the parasite’s genetic material into the body, and this DNA segment triggers the immune system to generate antibodies and activate immune cells to fight the infection.^63^ One of the main segments studied for this platform is cruzipain, identified as a key target antigen.

Cruzipain is a major cysteine protease of *T. cruzi* that plays a central role in parasite invasion, immune evasion and tissue damage during Chagas disease. It is crucial for parasite replication and pathogenesis. Notably, multiple cruzipain subtypes have been described, especially those from Family II, which are preferentially expressed during the mammalian stages of the parasite (amastigotes and trypomastigotes). These subtypes contain distinct immunogenic epitopes capable of eliciting broad T cell and antibody responses, making them promising candidates for vaccine design.^64^

A notable example of this platform is the oral bivalent vaccine based on cruzipain and its natural inhibitor, chagasin, delivered via *Salmonella enterica* as a DNA vector.^65^ This formulation has shown significant immunotherapeutic potential in murine models by inducing strong cellular and humoral immune responses, including elevated IFN-γ production, reduced parasitic burden and decreased tissue damage. Its oral administration route and low production cost make it particularly suitable for large-scale vaccination programs in endemic regions.^65^ These encouraging results support its candidacy for clinical trials and highlight its potential use in combination with trypanocidal drugs to enhance treatment efficacy and reduce the risk of congenital transmission of *T. cruzi*.^65^

Accordingly, in silico analyses and experimental data from murine models indicate that immunization with cruzipain elicits robust Th1-type immune responses.^64,66^ Moreover, this vaccine candidate stimulates specific IgG isotypes, which target key epitopes of cruzipain, particularly near or within its active site.^66^ When these IgG antibodies bind close to the active site where critical residues such as Cys25, His159 and Asn175 reside they inhibit cruzipain’s enzymatic activity by blocking its interaction with natural substrates.^64^ This prevents cruzipain from degrading host proteins and interferes with the parasite’s ability to evade the immune system.^64^ Additionally, the IgG antibodies can activate the classical complement pathway, promoting parasite destruction through complement-mediated lysis via the MAC or by enhancing phagocytosis.^23^ These immune mechanisms collectively contribute to the vaccine’s protective effect against *T. cruzi* infection.

## Recombinant protein vaccines

The development of vaccines against *T. cruzi*, the causative agent of Chagas disease, has advanced significantly thanks to the use of parasite-specific recombinant proteins. These strategies aim to induce immune responses that could be effective in both the acute and chronic phases of infection by coordinating humoral and cellular immunity. Below, some promising vaccine candidates based on recombinant proteins are described.

Other vaccine candidates against *T. cruzi* include TSA-1-C4 and Tc24-C4. In in vitro macrophage cultures, these recombinant proteins induce the release of nitric oxide and hydrogen peroxide, as well as pro-inflammatory cytokines such as TNF-α, IL-1β and IL-6, along with immunoregulatory cytokines like IL-10. The expression of these cytokines, particularly IL-6, can promote the differentiation of CD4⁺ T cells into Th17 effector cells. Additionally, IL-6 may support isotype switching in plasma cells toward IgG1–IgG3 subclasses, which could possess neutralizing activity against the parasite. Moreover, these peptides have been shown to activate Tc1 CD8⁺ T cells, enabling them to recognize and eliminate *T. cruzi*-infected cells.^67^ Therefore, the promising results observed in murine models support the advancement of TSA-1-C4 and Tc24-C4 to phase I clinical trials in humans.

In this context, Teh-Poot et al.^68^ designed a recombinant protein that combines conserved epitopes from different *T. cruzi* lineages, which exhibit high affinity for the HLA-A*02:01 allele, commonly found in Latin American populations. This protein was expressed in *Escherichia coli*, purified and functionally evaluated through ex vivo assays using peripheral blood mononuclear cells from patients with Chagas disease. The results demonstrated a robust immune response, characterized by elevated IFN-γ production, which was higher than that induced by mixtures of individual epitopes. This suggests that the recombinant protein format enhances antigen presentation and promotes more effective activation of CD8+ T cells. The activation of these CD8+ T cells with a Tc1 effector profile may play a crucial role in controlling the infection, as they can recognize *T. cruzi* epitopes presented on infected cells and induce their destruction through the release of perforins and granzymes, leading to osmotic lysis. Furthermore, as a multiepitope recombinant protein capable of inducing a Th1-type immune response, it may also promote the production of neutralizing IgG antibodies against the selected *T. cruzi* epitopes. This could help prevent or reduce the risk of infection, supporting the potential of this construct as a promising vaccine candidate against Chagas disease.

A promising approach was conducted in mice by Masip et al.^69^ involving a vaccine composed of recombinant proteins from the *T. cruzi*-specific TcTASV family. This vaccine combines aluminum hydroxide-adjuvanted proteins with a recombinant baculovirus displaying a TcTASV antigen on its capsid. The formulation elicits a robust Th1-biased immune response, characterized by elevated IFN-γ production and a predominance of the IgG2a isotype, both of which are essential for effective control of intracellular parasites. Additionally, the vaccine induces functional antibodies capable of mediating complement-dependent lysis of trypomastigotes, thereby disrupting the early stages of infection. It also activates TcTASV-A–specific CD8+ T cells and TcTASV-C–specific CD4+/IFN-γ+ T cells, which contribute to the clearance of infected host cells. Consequently, the vaccine demonstrates protective efficacy during both the acute and chronic phases of infection. Importantly, it prevents disease reactivation following immunosuppression and provides complete protection against lethal reinfection, highlighting its ability to generate robust and long-lasting immunological memory.

## RNA vaccines

The emergence of SARS-CoV-2 opened the door to a new vaccine platform based on messenger RNA (mRNA),^70^ capable of inducing a specific immune response against selected segments of target microorganisms. In the context of Chagas disease, a therapeutic mRNA vaccine targeting the Tc24 and ASP-2 antigens of *T. cruzi* has demonstrated a robust and durable immune response in murine models.^71^ This bivalent formulation stimulates the production of key pro-inflammatory cytokines essential for infection control, such as IFN-γ, TNF-α and IL-2, as well as regulatory cytokines like IL-4 and IL-10, showing a balanced Th1/Th2 response.^71^ The sustained activation of these cytokines in splenocytes up to 126 d postimmunization indicates the generation of prolonged immunological memory, surpassing that observed with monovalent formulations.^71^ This coordinated immune response significantly contributes to reducing parasite burden and cardiac inflammation during the chronic phase of the disease, highlighting the potential of mRNA vaccines as a promising therapeutic strategy against Chagas disease.^71^

## Conclusions and future directions

*Trypanosoma cruzi* continues to represent a major public health threat, particularly in endemic regions of Latin America, with increasing global relevance due to migration and environmental changes. The parasite’s ability to evade host immune responses through diverse and complex mechanisms, including disruption of innate signaling, complement inhibition and immune tolerance modulation, highlights the urgent need for innovative strategies. A deeper understanding of these evasion tactics is essential for the development of effective interventions. Moving forward, integrating this immunological knowledge into vaccine development and therapeutic design will be crucial to achieving sustainable control and eventual elimination of Chagas disease.

Regarding vaccine development, multiple innovative platforms, including CRISPR-Cas9 attenuated vaccines, DNA-based vaccines, recombinant protein vaccines and mRNA vaccines, have shown promising results in preclinical studies. These strategies aim to induce balanced immune responses capable of reducing parasitic burden and preventing progression to chronic disease. However, it is important to note that most of these candidates are still in the preclinical stage, and significant efforts are needed to advance them through clinical trials and obtain regulatory approval for use in humans. Moreover, it is evident that combining multiple antigens and platforms, alongside integrated approaches that consider ecological and epidemiological factors, will be essential for successful vaccine development. Therefore, addressing *T. cruzi* infection requires not only a deep understanding of its immune evasion mechanisms, but also a multidisciplinary and transnational effort to develop sustainable and effective interventions ultimately aimed at controlling and eliminating this neglected tropical disease.

## Data Availability

Not applicable.
